# Discovery of rare ancestry-specific variants in the fetal genome that confer risk of preterm premature rupture of membranes (PPROM) and preterm birth

**DOI:** 10.1186/s12881-018-0696-4

**Published:** 2018-10-05

**Authors:** Bhavi P. Modi, Hardik I. Parikh, Maria E. Teves, Rewa Kulkarni, Jiang Liyu, Roberto Romero, Timothy P. York, Jerome F. Strauss

**Affiliations:** 1 Department of Human and Molecular Genetics, Virginia Commonwealth University, Richmond, VA USA; 2 Department of Microbiology and Immunology, Virginia Commonwealth University, Richmond, VA USA; 3 Department of Obstetrics and Gynecology, Virginia Commonwealth University School of Medicine, Sanger Hall 11-029, 1101 East Marshall Street, Richmond, VA 23298 USA; 4 Perinatology Research Branch, Eunice Kennedy Shriver National Institute of Child Health and Human Development, NIH, Bethesda, MD USA; 5 Perinatology Research Branch, Eunice Kennedy Shriver National Institute of Child Health and Human Development, NIH, Detroit, MI USA

**Keywords:** Preterm premature rupture of membranes, Defensin β1, Mannose binding lectin-2, Methyltransferase like 7B, Whole exome sequencing

## Abstract

**Background:**

Preterm premature rupture of membranes (PPROM) is the leading identifiable cause of preterm birth, a complication that is more common in African Americans. Attempts to identify genetic loci associated with preterm birth using genome-wide association studies (GWAS) have only been successful with large numbers of cases and controls, and there has yet to be a convincing genetic association to explain racial/ethnic disparities. Indeed, the search for ancestry-specific variants associated with preterm birth has led to the conclusion that spontaneous preterm birth could be the consequence of multiple rare variants. The hypothesis that preterm birth is due to rare genetic variants that would go undetected in standard GWAS has been explored in the present study. The detection and validation of these rare variants present challenges because of the low allele frequency. However, some success in the identification of fetal loci/genes associated with preterm birth using whole genome sequencing and whole exome sequencing (WES) has recently been reported. While encouraging, this is currently an expensive technology, and methods to leverage the sequencing data to quickly identify and cost-effectively validate variants are needed.

**Methods:**

We developed a WES data analysis strategy based on neonatal genomic DNA from PPROM cases and term controls that was unencumbered by preselection of candidate genes, and capable of identifying variants in African Americans worthy of focused evaluation to establish statistically significant associations.

**Results:**

We describe this approach and the identification of damaging nonsense variants of African ancestry in the *DEFB1* and *MBL2* genes that encode anti-microbial proteins that presumably defend the fetal membranes from infectious agents. Our approach also enabled us to rule out a likely contribution of a predicted damaging nonsense variant in the *METTL7B* gene.

**Conclusions:**

Our findings support the notion that multiple rare population-specific variants in the fetal genome contribute to preterm birth associated with PPROM.

**Electronic supplementary material:**

The online version of this article (10.1186/s12881-018-0696-4) contains supplementary material, which is available to authorized users.

## Background

There are significant disparities in preterm birth rates in the United States, with African Americans experiencing an increased burden [1, 2]. Delivery after preterm premature rupture of the membranes (PPROM) is the leading identifiable cause of spontaneous preterm birth, and PPROM is more common in African-Americans. PPROM is believed to be caused, in part, by infection and inflammation, presumably incited by microbes ascending from the vagina, resulting in the release of pro-inflammatory cytokines and the activation of matrix-degrading proteases that breakdown the collagens that give the fetal membranes their tensile strength, resulting in unscheduled rupture [3–5].

Twin studies have revealed that both fetal and maternal genetic factors contribute to gestational age at delivery, but there is uncertainty about the roles played by specific fetal and maternal genes. Attempts to identify genetic loci associated with gestational age at delivery and preterm birth using genome-wide association studies (GWAS) have only been successful with large numbers of cases and controls (see reference [5] for a review). Moreover, these studies have not identified genes that could account for increased preterm births in African-Americans. Efforts to identify ancestry-specific variants using GWAS approaches have led to the conclusion that spontaneous preterm birth is likely to be the consequence of multiple common variants or rare variants not easily detected by GWAS [6]. This is not a surprising conclusion since GWAS is based the “common disease-common variant” hypothesis, positing that a significant proportion of the variance of common diseases are attributable to DNA variants that are present in > 1–5% of the population, and that there are many of these DNA variants, each contributing a small amount to the total risk to a particular disease [5].

As noted above, an alternative hypothesis is that diseases are associated with rare genetic variants that have relatively larger effect sizes that would go undetected in standard GWAS. The detection and validation of these rare variants presents challenges because of the low allele frequency. Some success in the identification of fetal loci/genes associated with preterm birth using whole genome sequencing and whole exome sequencing (WES) has recently been reported [7–9]. While encouraging, this is currently expensive technology and methods to leverage the sequencing data to quickly identify and cost-effectively validate variants are needed.

We recently pursued the approach of searching for rare variants in fetal genes that could contribute to risk of PPROM, employing WES to identify the burden of damaging mutations in African-American fetal (neonatal) samples [8, 9]. In one of our studies, our analysis of the WES data focused on genes that either negatively regulate the innate immune response or which encode proteins that protect the host against microbes and their noxious products. Rather than utilizing a prospective candidate gene filter, we decided to develop a WES analysis plan that was not encumbered by preselection and capable of identifying rare damaging variants in African-Americans worth focused evaluation to establish statistically significant association and the mechanism(s) underlying the mutation effect. We hoped to establish a cost effective simple process that could be applied to modest sample sizes.

## Methods

### Subjects

The subjects in the discovery WES (76 PPROM cases and 43 term controls) and initial confirmatory targeted genotyping for *DEFB1* and *MBL2* nonsense variants (188 PPROM cases and 175 term controls) have been previously described [8, 9]. The *METTL7B* SNPs were evaluated with the WES cohort. They were neonates born of self-reported African-American women. Term controls consisted of neonates born from uncomplicated singleton pregnancies (> 37 weeks gestation). PPROM cases were from singleton pregnancies prior to 37 weeks of completed gestation. The diagnosis of membrane rupture was based on pooling of amniotic fluid in the vagina, amniotic fluid ferning patterns and a positive nitrazine test. Women with multiple gestations, fetal anomalies, trauma, connective tissue diseases and medical complications of pregnancy requiring induction of labor were excluded as previously described [8, 9].

The previously published analysis of the *DEFB1* and *MBL2* nonsense variants included the WES cohort and initial confirmatory targeted genotyping cohort described above [9]. In the present study the *METTL7B* SNPs were evaluated with the original WES cohort. In addition, we performed targeted genotyping for the *DEFB1* and *MBL2* nonsense variants on 119 PPROM cases and 199 controls not previously reported. The subjects were recruited from the same populations as the previously reported cohorts using identical inclusion and exclusion criteria. Ninety-four of these PPROM cases and 94 term controls were used for genotyping *METTL7B* SNPs.

### Whole exome sequencing and genotyping

The methods used for WES (50-100X coverage) and analysis of the sequencing data have been described in detail in previous publications [8, 9]. With the number of PPROM cases (76), we had 78% power to detect variants with a minor allele frequency of 0.005. Targeted genotyping was performed on the Agena (previously Sequenom) MassArray iPLEX platform [8, 9]. The primers used for *METTL7B* genotyping are presented in Additional file 1: Table S1. Only high confidence genotype calls were included in the analysis.

#### Estimation of African ancestry

To reduce the potential risk that population stratification biased the genetic association tests, the percent African ancestry of the PPROM neonates and term control neonates was determined using ancestry-informative markers as previously described [8, 9]. No significant differences in the percentage of African ancestry were found between PPROM cases and term controls (Means +/− S.D.; West African ancestry: PPROM cases: 0.695 +/− 0.073 (mean + S.D.); Term controls 0.698 +/− 0.087 (*p* > 0.10)) [9].

### RT-PCR analysis

Detection of DEFB1 and METTL7B transcripts in fetal membrane RNA (1 μg) was accomplished by PCR as previously described [8]. The primers used for DEFB1 transcript amplification were forward 5’-CTGAAATCCTGGGTGTTGCC-3′ and reverse 5’-CTTCTGGTCACTCCCAGCTC-3′. The primers used for METTL7B transcript amplification were forward 5’-ACCTGCCTAGACCCAAATCC-3′ and reverse 5′- TTATTTGACAGCCTTTCCCATGA-3′. In both cases, PCR was run for 40 cycles and amplified bands were sequence verified to be the cognate transcripts.

### Selection strategy

We developed the following simple screening method for analyzing the WES data: 1) Identify predicted damaging nonsense variants (gnomAD “high confidence”) present only in PPROM cases in the WES discovery panel; 2) Validate the nonsense variants by Sanger sequencing; 3) Verify it is a rare variant (minor allele frequency < 0.01) based on the genome aggregation database (gnomAD: http://gnomad.broadinstitute.org/); 4) Determine whether the variant/mutation is of African ancestry using a public database (gnomAD); 5) Determine whether the gene is under selective pressure, consistent with an essential role in a biological or pathophysiological process, by a literature review (PubMed: https://www.ncbi.nlm.nih.gov/pubmed/); 6) Assess whether heterozygous variants could potentially cause a biological effect by altering the expression level or activity of mature protein; 7) Determine whether the gene harboring the nonsense variant is expressed in fetal membranes; 8) Evaluate whether the gene could play a role in the existing pathophysiological concepts of PPROM from the literature; and 9) Conduct follow-up genotyping of the nonsense variant in independent cohorts to test the association of the identified variant with PPROM.

### Statistical analysis

The minor allele frequencies for the *DEFB1* and *MBL2* nonsense variants examined in this report would require a very large number of PPROM cases and controls for an association study to achieve a power of 0.8 and a *p* value = 0.05. Therefore, we combined all WES and genotyping data reported previously [9] with the results of the genotyping of the additional subjects for each nonsense variant in the analysis. Finding no genetic association with these samples sizes cannot rule out an association. However, the discovery of significant associations, albeit in a study of limited power, does not negate the findings, with the caveat that significant findings from low powered studies may not always replicate.

Associations were examined for statistical significance using Fisher’s Exact test (1-tailed) to determine whether the nonsense variant was overrepresented in PPROM cases. Nominal *p* values are reported. Correcting for multiple tests (Bonferroni adjustment) a, p value of < 0.017 would be considered the threshold for statistical significance, which was met for the *DEFB1* and *MBL2* nonsense variants studied.

## Results

We detected more than 800 different nonsense variants (stop gain, stop loss, and start loss) in the discovery WES sample of PPROM cases and term controls, approximately 33% of which were unique to PPROM, the majority of which occurred in only one PPROM case, and 30% of the variant types were unique to term controls, with the majority occurring in one term control (Table 1) The remaining approximately one third of the nonsense variants occurred both in PPROM cases and controls, and not unexpectedly were nonsense variants with the highest allele frequency, suggesting that these variants might be tolerated and do not contribute to PPROM risk. Most of these nonsense variants have been previously detected in the human genome. More than 1400 coding sequence frameshift variants and splicing variants, predicted to be or possibly damaging were detected. Since a number of these variants were not previously known, it is uncertain whether they reflect sequencing errors in the WES. We suspect the latter since Sanger sequencing of a number of the DNA samples failed to confirm frameshift mutations. Consequently, we did not include the predicted damaging frameshift and splicing mutations in our screening paradigm.

**Table 1:** Nonsense Variants in PPROM Cases Identified by WES

Gene	Transcript	dbSNP141	Chr	Start	End	Ref	Obs	Cases^a^	Controls^a^
One variant allele in PPROM Cohort									
ABCA7	NM_019112.3:p.Arg285^a^/c.853A > T	rs77403558	chr19	1043395	1043395	A	T	0,1,1	0,0,0
ABCB5	NM_001163941.1:p.Lys626^a^/c.1876A > T	rs76179099	chr7	20725325	20725325	A	T	0,1,1	0,0,0
ABCB5	NM_001163941.1:p.Gln1195^a^/c.3583C > T	rs146527949	chr7	20795056	20795056	C	T	0,1,1	0,0,0
ABCC3	NM_003786.3:p.Gln132^a^/c.394C > T	rs201830141	chr17	48734452	48734452	C	T	0,1,1	0,0,0
ACAD11	NM_032169.4:p.Glu13^a^/c.37G > T	rs151048899	chr3	132378559	132378559	C	A	0,1,1	0,0,0
ACOXL	NM_001142807.1:p.Gly577^a^/c.1729G > T	rs189429375	chr2	111875379	111875379	G	T	0,1,1	0,0,0
ACTN2	NM_001103.3:p.Glu120^a^/c.358G > T		chr1	236882310	236882310	G	T	0,1,1	0,0,0
ADAMTS14	NM_139155.2:p.Arg317^a^/c.949C > T	rs199886417	chr10	72489128	72489128	C	T	0,1,1	0,0,0
AHCTF1	NM_015446.4:p.Ser1120^a^/c.3359C > A		chr1	247030561	247030561	G	T	0,1,1	0,0,0
ALDH3B1	NM_000694.3:p.Trp135^a^/c.405G > A	rs375063489	chr11	67786650	67786650	G	A	0,1,1	0,0,0
ANGPTL7	NM_021146.3:p.Trp188^a^/c.563G > A	rs145750805	chr1	11253722	11253722	G	A	0,1,1	0,0,0
ANKRD65	NM_001243535.1:p.Trp108^a^/c.323G > A		chr1	1354816	1354816	C	T	0,1,1	0,0,0
AP3B1	NM_003664.4:p.Arg407^a^/c.1219C > T		chr5	77461445	77461445	G	A	0,1,1	0,0,0
AP5Z1	NM_014855.2:p.Trp441^a^/c.1322G > A	rs373919408	chr7	4827275	4827275	G	A	0,1,1	0,0,0
ATP2C2	NM_001286527.2:p.Tyr106^a^/c.318C > G		chr16	84438841	84438841	C	G	0,1,1	0,0,0
AZU1	NM_001700.3:p.Arg236^a^/c.706C > T	rs112572343	chr19	831827	831827	C	T	0,1,1	0,0,0
BCL2L12	NM_138639.1:p.Tyr44^a^/c.132 T > G	rs141156787	chr19	50169212	50169212	T	G	0,1,1	0,0,0
BCLAF1	NM_014739.2:p.Arg298^a^/c.892C > T	rs138333275	chr6	136599127	136599127	G	A	0,1,1	0,0,0
BPIFB6	NM_174897.2:p.Tyr434^a^/c.1302C > A	rs140595029	chr20	31631146	31631146	C	A	0,1,1	0,0,0
C12orf40	NM_001031748.2:p.Gln568^a^/c.1702C > T	rs140530325	chr12	40114796	40114796	C	T	0,1,1	0,0,0
C12orf42	NM_001099336.2:p.Glu13^a^/c.37G > T	rs202081871	chr12	103872168	103872168	C	A	0,1,1	0,0,0
C12orf56	NM_001170633.1:p.Lys269^a^/c.805A > T	rs201295265	chr12	64712444	64712444	T	A	0,1,1	0,0,0
C15orf32	NM_153040.2:p.Lys30^a^/c.88A > T	rs115999940	chr15	93015466	93015466	A	T	0,1,1	0,0,0
C18orf54	NM_001288980.1:p.Ser530^a^/c.1589C > A	rs148065410	chr18	51904603	51904603	C	A	0,1,1	0,0,0
C20orf173	NM_001145350.1:p.Arg36^a^/c.106C > T	rs141795719	chr20	34117097	34117097	G	A	0,1,1	0,0,0
C20orf78	NM_001242671.1:p.Trp115^a^/c.345G > A	rs146528664	chr20	18790531	18790531	C	T	0,1,1	0,0,0
C9orf50	NM_199350.3:p.Gln406^a^/c.1216C > T	rs374957154	chr9	132374706	132374706	G	A	0,1,1	0,0,0
CACNA1A	NM_023035.2:p.Arg1918^a^/c.5752C > T	rs16044	chr19	13325415	13325415	G	A	0,1,1	0,0,0
CAMK4	NM_001744.4:p.Glu439^a^/c.1315G > T		chr5	110820057	110820057	G	T	0,1,1	0,0,0
CAPN11	NM_007058.3:p.Gln133^a^/c.397C > T	rs189429774	chr6	44137700	44137700	C	T	0,1,1	0,0,0
CARD6	NM_032587.3:p.Leu560^a^/c.1679 T > G	rs150487186	chr5	40853113	40853113	T	G	0,1,1	0,0,0
CCDC153	NM_001145018.1:p.Arg42^a^/c.124C > T	rs77842401	chr11	119065645	119065645	G	A	0,1,1	0,0,0
CCDC168	NM_001146197.1:p.Tyr6396^a^/c.19188 T > G	rs73587211	chr13	103383859	103383859	A	C	0,1,1	0,0,0
CCDC3	NM_031455.3:p.Ser188^a^/c.563C > A	rs150029612	chr10	12940666	12940666	G	T	0,1,1	0,0,0
CCDC57	NM_198082.2:p.Arg676^a^/c.2026C > T	rs201336748	chr17	80121090	80121090	G	A	0,1,1	0,0,0
CCDC60	NM_178499.3:p.Arg520^a^/c.1558C > T	rs78597191	chr12	119978425	119978425	C	T	0,1,1	0,0,0
CCT8L2	NM_014406.4:p.Trp320^a^/c.959G > A	rs144853652	chr22	17072482	17072482	C	T	0,1,1	0,0,0
CD1A	NM_001763.2:p.Arg249^a^/c.745C > T	rs149659983	chr1	158226716	158226716	C	T	0,1,1	0,0,0
CEP135	NM_025009.4:p.Gln824^a^/c.2470C > T		chr4	56876034	56876034	C	T	0,1,1	0,0,0
CETP	NM_000078.2:p.Glu133^a^/c.397G > T		chr16	57003551	57003551	G	T	0,1,1	0,0,0
CFHR4	NM_001201550.2:p.Arg41^a^/c.121A > T	rs199547603	chr1	196871610	196871610	A	T	0,1,1	0,0,0
CHD1L	NM_004284.4:p.Arg611^a^/c.1831C > T		chr1	146756149	146756149	C	T	0,1,1	0,0,0
CLEC2A	NM_001130711.1:p.Trp137^a^/c.411G > A	rs142033208	chr12	10066279	10066279	C	T	0,1,1	0,0,0
CLEC4A	NM_016184.3:p.Trp176^a^/c.528G > A	rs115176426	chr12	8289461	8289461	G	A	0,1,1	0,0,0
COL6A5	NM_001278298.1:p.Gln1184^a^/c.3550C > T	rs115380050	chr3	130114290	130114290	C	T	0,1,1	0,0,0
CPA3	NM_001870.2:p.Arg178^a^/c.532C > T	rs145845146	chr3	148597632	148597632	C	T	0,1,1	0,0,0
CPA6	NM_020361.4:p.Arg311^a^/c.931C > T	rs139145929	chr8	68346383	68346383	G	A	0,1,1	0,0,0
CPO	NM_173077.2:p.Trp102^a^/c.305G > A	rs138166151	chr2	207823062	207823062	G	A	0,1,1	0,0,0
CTSV	NM_001201575.1:p.Arg96^a^/c.286C > T		chr9	99799644	99799644	G	A	0,1,1	0,0,0
CYP1A2	NM_000761.4:p.Tyr495^a^/c.1485C > A	rs143193369	chr15	75047363	75047363	C	A	0,1,1	0,0,0
DACT2	NM_001286351.1:p.Ser264^a^/c.791C > G		chr6	168694817	168694817	G	C	0,1,1	0,0,0
DCDC5	NM_020869.3:p.Arg864^a^/c.2590C > T	rs200380430	chr11	30900214	30900214	G	A	0,1,1	0,0,0
DCHS2	NM_017639.3:p.Arg1343^a^/c.4027C > T	rs150179829	chr4	155226252	155226252	G	A	0,1,1	0,0,0
DEFB1	NM_005218.3:c.111C>A	rs5743490	chr8	6728299	6728299	G	T	0,1,1	0,0,0
DLEC1	NM_007337.2:p.Cys1696^a^/c.5088C > A	rs201487599	chr3	38163847	38163847	C	A	0,1,1	0,0,0
DNAH10	NM_207437.3:p.Arg3898^a^/c.11692C > T		chr12	124411308	124411308	C	T	0,1,1	0,0,0
DNAH14	NM_001373.1:p.Glu19^a^/c.55G > T	rs61745064	chr1	225140459	225140459	G	T	0,1,1	0,0,0
DRD4	NM_000797.3:p.Glu62^a^/c.184G > T		chr11	637488	637488	G	T	0,1,1	0,0,0
DTHD1	NM_001170700.2:p.Trp678^a^/c.2034G > A	rs149895631	chr4	36345134	36345134	G	A	0,1,1	0,0,0
EBLN2	NM_018029.3:p.Tyr164^a^/c.492 T > G	rs2231925	chr3	73111724	73111724	T	G	0,1,1	0,0,0
ECHDC2	NM_001198961.1:p.Trp11^a^/c.33G > A	rs368731634	chr1	53387313	53387313	C	T	0,1,1	0,0,0
EDDM3A	NM_006683.4:p.Arg43^a^/c.127C > T	rs138978934	chr14	21215866	21215866	C	T	0,1,1	0,0,0
EFCAB13	NM_152347.4:p.Arg236^a^/c.706C > T	rs78865644	chr17	45438788	45438788	C	T	0,1,1	0,0,0
EFCAB13	NM_152347.4:p.Lys433^a^/c.1297A > T	rs74969489	chr17	45452257	45452257	A	T	0,1,1	0,0,0
EGF	NM_001963.4:p.Gln1095^a^/c.3283C > T	rs138244768	chr4	110925770	110925770	C	T	0,1,1	0,0,0
EIF3J	NM_003758.3:p.Glu192^a^/c.574G > T		chr15	44852449	44852449	G	T	0,1,1	0,0,0
ELOVL5	NM_001242830.1:p.Gly246^a^/c.736G > T	rs41273878	chr6	53133964	53133964	C	A	0,1,1	0,0,0
ELOVL5	NM_001242828.1:p.Gln102^a^/c.304C > T	rs150583340	chr6	53152683	53152683	G	A	0,1,1	0,0,0
ELP4	NM_001288725.1:p.Gln385^a^/c.1153C > T		chr11	31784965	31784965	C	T	0,1,1	0,0,0
ENGASE	NM_001042573.2:p.Arg352^a^/c.1054C > T	rs149186913	chr17	77079117	77079117	C	T	0,1,1	0,0,0
EOGT	NM_001278689.1:p.Lys188^a^/c.562A > T	rs116711473	chr3	69053587	69053587	T	A	0,1,1	0,0,0
EPB41L4A	NM_022140.3:p.Arg348^a^/c.1042C > T	rs368151776	chr5	111570376	111570376	G	A	0,1,1	0,0,0
EVC2	NM_147127.4:p.Lys342^a^/c.1024A > T		chr4	5664955	5664955	T	A	0,1,1	0,0,0
EVPL	NM_001988.2:p.Gln938^a^/c.2812C > T	rs151046085	chr17	74006474	74006474	G	A	0,1,1	0,0,0
EVPLL	NM_001145127.1:p.Trp209^a^/c.627G > A	rs182498101	chr17	18286454	18286454	G	A	0,1,1	0,0,0
EYS	NM_001292009.1:p.Trp2090^a^/c.6270G > A		chr6	64940639	64940639	C	T	0,1,1	0,0,0
FAM179A	NM_199280.2:p.Arg162^a^/c.484C > T	rs183676260	chr2	29225458	29225458	C	T	0,1,1	0,0,0
FAM187B	NM_152481.1:p.Trp231^a^/c.693G > A	rs35001809	chr19	35718891	35718891	C	T	0,1,1	0,0,0
FAM200A	NM_145111.3:p.Leu61^a^/c.182 T > G		chr7	99145849	99145849	A	C	0,1,1	0,0,0
FAM227B	NM_152647.2:p.Arg5^a^/c.13C > T	rs140471517	chr15	49907356	49907356	G	A	0,1,1	0,0,0
FAM60A	NM_001135811.1:p.Ser70^a^/c.209C > A		chr12	31448187	31448187	G	T	0,1,1	0,0,0
FASTKD1	NM_024622.4:p.Ser768^a^/c.2303C > G	rs34291832	chr2	170387886	170387886	G	C	0,1,1	0,0,0
FBXL21	NM_012159.4:p.Trp72^a^/c.215G > A	rs148275750	chr5	135272498	135272498	G	A	0,1,1	0,0,0
FBXO48	NM_001024680.1:p.Glu134^a^/c.400G > T	rs148116960	chr2	68691409	68691409	C	A	0,1,1	0,0,0
FCRL6	NM_001004310.2:p.Gln406^a^/c.1216C > T		chr1	159785362	159785362	C	T	0,1,1	0,0,0
FDXR	NM_001258013.2:p.Gln66^a^/c.196C > T	rs187001043	chr17	72868325	72868325	G	A	0,1,1	0,0,0
FMO1	NM_001282692.1:p.Arg506^a^/c.1516C > T	rs60639054	chr1	171254588	171254588	C	T	0,1,1	0,0,0
FREM3	NM_001168235.1:p.Gln166^a^/c.496C > T		chr4	144621333	144621333	G	A	0,1,1	0,0,0
FTCD	NM_006657.2:p.Arg71^a^/c.211C > T	rs8133955	chr21	47574090	47574090	G	A	0,1,1	0,0,0
FXYD3	NM_001136007.1:p.Arg45^a^/c.133C > T		chr19	35610131	35610131	C	T	0,1,1	0,0,0
GAL3ST4	NM_024637.4:p.Trp289^a^/c.867G > A	rs147809354	chr7	99758145	99758145	C	T	0,1,1	0,0,0
GCAT	NM_001171690.1:p.Trp2^a^/c.5G > A	rs202183602	chr22	38203979	38203979	G	A	0,1,1	0,0,0
GNB3	NM_002075.3:p.Lys89^a^/c.265A > T		chr12	6952399	6952399	A	T	0,1,1	0,0,0
GP6	NM_001083899.2:p.Trp429^a^/c.1287G > A	rs74697203	chr19	55526026	55526026	C	T	0,1,1	0,0,0
GPR148	NM_207364.2:p.Arg81^a^/c.241C > T	rs140681574	chr2	131486965	131486965	C	T	0,1,1	0,0,0
GSG1	NM_001080555.2:p.Arg160^a^/c.478A > T		chr12	13241799	13241799	T	A	0,1,1	0,0,0
GTDC1	NM_001284238.1:p.Trp9^a^/c.26G > A	rs145066970	chr2	144934759	144934759	C	T	0,1,1	0,0,0
GUCA1C	NM_005459.3:p.Glu18^a^/c.52G > T	rs143174402	chr3	108672558	108672558	C	A	0,1,1	0,0,0
HEPHL1	NM_001098672.1:p.Trp96^a^/c.288G > A		chr11	93778956	93778956	G	A	0,1,1	0,0,0
HERC6	NM_017912.3:p.Gln1021^a^/c.3061C > T	rs4413373	chr4	89363604	89363604	C	T	0,1,1	0,0,0
HIST1H1T	NM_005323.3:p.Arg168^a^/c.502A > T	rs35191055	chr6	26107820	26107820	T	A	0,1,1	0,0,0
HKDC1	NM_025130.3:p.Trp721^a^/c.2163G > A	rs147565138	chr10	71018662	71018662	G	A	0,1,1	0,0,0
HMCN2	NM_001291815.1:p.Arg3622^a^/c.10864C > T		chr9	133281539	133281539	C	T	0,1,1	0,0,0
HRG	NM_000412.3:p.Glu294^a^/c.880G > T	rs140336956	chr3	186394974	186394974	G	T	0,1,1	0,0,0
HSD17B14	NM_016246.2:p.Arg79^a^/c.235C > T	rs139341223	chr19	49335965	49335965	G	A	0,1,1	0,0,0
IQCE	NM_001287499.1:p.Ser366^a^/c.1097C > A	rs367705543	chr7	2629593	2629593	C	A	0,1,1	0,0,0
ITGA10	NM_003637.3:p.Arg313^a^/c.937C > T		chr1	145532484	145532484	C	T	0,1,1	0,0,0
JMJD7-PLA2G4B	NM_005090.3:p.Arg486^a^/c.1456C > T	rs199962342	chr15	42135893	42135893	C	T	0,1,1	0,0,0
KCNJ16	NM_001291622.1:p.Gly168^a^/c.502G > T		chr17	68128625	68128625	G	T	0,1,1	0,0,0
KCNJ16	NM_001291622.1:p.Arg337^a^/c.1009C > T	rs142625269	chr17	68129132	68129132	C	T	0,1,1	0,0,0
KCNJ18	NM_001194958.2:p.Arg399^a^/c.1195C > T	rs144702327	chr17	21319849	21319849	C	T	0,1,1	0,0,0
KCNU1	NM_001031836.2:p.Trp768^a^/c.2303G > A		chr8	36767025	36767025	G	A	0,1,1	0,0,0
KIAA0319L	NM_024874.4:p.Arg1019^a^/c.3055C > T		chr1	35900590	35900590	G	A	0,1,1	0,0,0
KIAA0753	NM_014804.2:p.Gln896^a^/c.2686C > T	rs149782904	chr17	6493199	6493199	G	A	0,1,1	0,0,0
KIF27	NM_017576.2:p.Arg1336^a^/c.4006C > T	rs371473677	chr9	86452116	86452116	G	A	0,1,1	0,0,0
KLHDC7A	NM_152375.2:p.Gln252^a^/c.754C > T	rs115859684	chr1	18808229	18808229	C	T	0,1,1	0,0,0
KLHDC9	NM_152366.4:p.Trp223^a^/c.669G > A	rs150493322	chr1	161069277	161069277	G	A	0,1,1	0,0,0
KLHL33	NM_001109997.2:p.Arg230^a^/c.688C > T		chr14	20898147	20898147	G	A	0,1,1	0,0,0
KLK4	NM_004917.3:p.Trp153^a^/c.458G > A	rs104894704	chr19	51411852	51411852	C	T	0,1,1	0,0,0
KLRF1	NM_016523.2:p.Trp128^a^/c.383G > A		chr12	9994456	9994456	G	A	0,1,1	0,0,0
LFNG	NM_001166355.1:p.Ser27^a^/c.80C > A	rs372947239	chr7	2552823	2552823	C	A	0,1,1	0,0,0
LHX4	NM_033343.3:p.Gln29^a^/c.85C > T		chr1	180217428	180217428	C	T	0,1,1	0,0,0
LILRB2	NM_005874.4:p.Glu161^a^/c.481G > T	rs370409653	chr19	54783377	54783377	C	A	0,1,1	0,0,0
LTBP4	NM_001042544.1:p.Trp1176^a^/c.3527G > A	rs35079932	chr19	41128415	41128415	G	A	0,1,1	0,0,0
LY9	NM_002348.3:p.Arg478^a^/c.1432C > T	rs145664274	chr1	160788097	160788097	C	T	0,1,1	0,0,0
MAFA	NM_201589.3:p.Lys346^a^/c.1036A > T		chr8	144511541	144511541	T	A	0,1,1	0,0,0
MALRD1	NM_001142308.2:p.Gln888^a^/c.2662C > T		chr10	19498280	19498280	C	T	0,1,1	0,0,0
MARK1	NM_001286124.1:p.Arg548^a^/c.1642C > T		chr1	220825398	220825398	C	T	0,1,1	0,0,0
MBL2	NM_000242.2:p.Glu210^a^c.628G > T	rs74754826	chr10	54528016	54528016	C	A	0,1,1	0,0,0
MCEMP1	NM_174918.2:p.Gln183^a^/c.547C > T	rs113286748	chr19	7743869	7743869	C	T	0,1,1	0,0,0
MDM1	NM_017440.4:p.Arg643^a^/c.1927C > T	rs147627177	chr12	68696445	68696445	G	A	0,1,1	0,0,0
MDN1	NM_014611.2:p.Glu4974^a^/c.14920G > T		chr6	90368430	90368430	C	A	0,1,1	0,0,0
METTL2A	NM_181725.3:p.Arg291^a^/c.871C > T	rs147656413	chr17	60522259	60522259	C	T	0,1,1	0,0,0
MGAT4D	NM_001277353.1:p.Lys372^a^/c.1114A > T		chr4	141372566	141372566	T	A	0,1,1	0,0,0
MIB1	NM_020774.3:p.Gln219^a^/c.655C > T		chr18	19358082	19358082	C	T	0,1,1	0,0,0
MIB2	NM_001170689.1:p.Gln739^a^/c.2215C > T	rs146481628	chr1	1565047	1565047	C	T	0,1,1	0,0,0
MLANA	NM_005511.1:p.Arg51^a^/c.151C > T		chr9	5897630	5897630	C	T	0,1,1	0,0,0
MLF1	NM_001195432.1:p.Arg194^a^/c.580C > T		chr3	158317881	158317881	C	T	0,1,1	0,0,0
MROH2A	NM_001287395.1:p.Gln944^a^/c.2830C > T		chr2	234723288	234723288	C	T	0,1,1	0,0,0
MST1R	NM_002447.2:p.Gln690^a^/c.2068C > T	rs61734381	chr3	49934828	49934828	G	A	0,1,1	0,0,0
MST1R	NM_002447.2:p.Lys621^a^/c.1861A > T	rs9819888	chr3	49935503	49935503	T	A	0,1,1	0,0,0
MUC12	NM_001164462.1:p.Arg171^a^/c.511C > T		chr7	100634355	100634355	C	T	0,1,1	0,0,0
MUC19	NM_173600.2:p.Arg7595^a^/c.22783C > T	rs183548726	chr12	40938465	40938465	C	T	0,1,1	0,0,0
MYOM3	NM_152372.3:p.Arg513^a^/c.1537C > T		chr1	24416105	24416105	G	A	0,1,1	0,0,0
NARR	NM_001256281.1:p.Gln177^a^/c.529C > T	rs140500150	chr17	27043980	27043980	G	A	0,1,1	0,0,0
NCOA1	NM_003743.4:p.Arg1122^a^/c.3364C > T		chr2	24964713	24964713	C	T	0,1,1	0,0,0
NGB	NM_021257.3:p.Gln11^a^/c.31C > T		chr14	77737250	77737250	G	A	0,1,1	0,0,0
NIPSNAP3A	NM_015469.1:p.Arg96^a^/c.286C > T	rs34856872	chr9	107515201	107515201	C	T	0,1,1	0,0,0
NKX1–2	NM_001146340.1:p.Trp4^a^/c.12G > A		chr10	126138501	126138501	C	T	0,1,1	0,0,0
NLRP12	NM_001277126.1:p.Arg1017^a^/c.3049C > T	rs35064500	chr19	54299165	54299165	G	A	0,1,1	0,0,0
NME3	NM_002513.2:p.Trp159^a^/c.477G > A	rs140703991	chr16	1820683	1820683	C	T	0,1,1	0,0,0
NUDT7	NM_001105663.2:p.Glu8^a^/c.22G > T	rs182579196	chr16	77756501	77756501	G	T	0,1,1	0,0,0
OAS1	NM_001032409.1:p.Arg73^a^/c.217C > T	rs147431531	chr12	113346377	113346377	C	T	0,1,1	0,0,0
OR10K1	NM_001004473.1:p.Tyr259^a^/c.777C > A	rs143219550	chr1	158436128	158436128	C	A	0,1,1	0,0,0
OR10R2	NM_001004472.1:p.Lys73^a^/c.217A > T		chr1	158449884	158449884	A	T	0,1,1	0,0,0
OR1G1	NM_003555.1:p.Ser95^a^/c.284C > G		chr17	3030562	3030562	G	C	0,1,1	0,0,0
OR2M4	NM_017504.1:p.Arg223^a^/c.667C > T	rs143728385	chr1	248402897	248402897	C	T	0,1,1	0,0,0
OR4M2	NM_001004719.2:p.Tyr177^a^/c.531C > G	rs148183880	chr15	22369106	22369106	C	G	0,1,1	0,0,0
OR5A1	NM_001004728.1:p.Arg125^a^/c.373C > T	rs150073749	chr11	59211014	59211014	C	T	0,1,1	0,0,0
OR5M11	NM_001005245.1:p.Tyr126^a^/c.378 T > A	rs17547284	chr11	56310356	56310356	A	T	0,1,1	0,0,0
OR8I2	NM_001003750.1:p.Tyr289^a^/c.867C > G	rs61887097	chr11	55861650	55861650	C	G	0,1,1	0,0,0
OR8U8	NM_001013356.2:p.Trp270^a^/c.810G > A	rs140673261	chr11	56143919	56143919	G	A	0,1,1	0,0,0
P2RY4	NM_002565.3:p.Trp348^a^/c.1043G > A	rs41310667	chrX	69478432	69478432	C	T	0,1,1	0,0,0
PADI2	NM_007365.2:p.Gln340^a^/c.1018C > T	rs142403504	chr1	17410253	17410253	G	A	0,1,1	0,0,0
PCDHGA10	NM_018913.2:p.Tyr331^a^/c.993 T > G		chr5	140793735	140793735	T	G	0,1,1	0,0,0
PCOLCE2	NM_013363.3:p.Arg204^a^/c.610C > T	rs143280691	chr3	142557712	142557712	G	A	0,1,1	0,0,0
PCSK6	NM_002570.4:p.Arg413^a^/c.1237C > T	rs77239269	chr15	101929740	101929740	G	A	0,1,1	0,0,0
PCSK9	NM_174936.3:p.Cys679^a^/c.2037C > A	rs28362286	chr1	55529215	55529215	C	A	0,1,1	0,0,0
PDE5A	NM_001083.3:p.Gln860^a^/c.2578C > T	rs140289122	chr4	120419806	120419806	G	A	0,1,1	0,0,0
PDIA6	NM_001282705.1:p.Arg13^a^/c.37C > T		chr2	10977695	10977695	G	A	0,1,1	0,0,0
PEG3	NM_001146184.1:p.Trp16^a^/c.48G > A		chr19	57335976	57335976	C	T	0,1,1	0,0,0
PEG3	NM_001146184.1:p.Lys14^a^/c.40A > T		chr19	57335984	57335984	T	A	0,1,1	0,0,0
PEX7	NM_000288.3:p.Leu292^a^/c.875 T > A	rs1805137	chr6	137219351	137219351	T	A	0,1,1	0,0,0
PIF1	NM_001286497.1:p.Glu49^a^/c.145G > T	rs75683534	chr15	65116390	65116390	C	A	0,1,1	0,0,0
PLA2R1	NM_007366.4:p.Cys1377^a^/c.4131 T > A	rs145354671	chr2	160801430	160801430	A	T	0,1,1	0,0,0
PLCD4	NM_032726.3:p.Arg117^a^/c.349C > T	rs146112514	chr2	219483469	219483469	C	T	0,1,1	0,0,0
PLCL2	NM_001144382.1:p.Arg1115^a^/c.3343C > T	rs149144281	chr3	17131374	17131374	C	T	0,1,1	0,0,0
POLR3B	NM_018082.5:p.Glu374^a^/c.1120G > T		chr12	106820993	106820993	G	T	0,1,1	0,0,0
PROSER3	NM_001039887.2:p.Arg439^a^/c.1315C > T	rs375920305	chr19	36259319	36259319	C	T	0,1,1	0,0,0
PRSS41	NM_001135086.1:p.Gln69^a^/c.205C > T		chr16	2849034	2849034	C	T	0,1,1	0,0,0
PSME1	NM_176783.2:p.Gln229^a^/c.685C > T	rs370880151	chr14	24607785	24607785	C	T	0,1,1	0,0,0
PSRC1	NM_001005290.3:p.Lys249^a^/c.745A > T	rs116389032	chr1	109823457	109823457	T	A	0,1,1	0,0,0
PTBP3	NM_001244898.1:p.Arg56^a^/c.166C > T	rs143872137	chr9	115038264	115038264	G	A	0,1,1	0,0,0
PTGDR	NM_000953.2:p.Trp48^a^/c.143G > A	rs41533946	chr14	52734675	52734675	G	A	0,1,1	0,0,0
PXDNL	NM_144651.4:p.Cys1258^a^/c.3774 T > A	rs117752382	chr8	52284560	52284560	A	T	0,1,1	0,0,0
PZP	NM_002864.2:p.Gln1168^a^/c.3502C > T	rs143616823	chr12	9309819	9309819	G	A	0,1,1	0,0,0
RABEP1	NM_004703.5:p.Arg270^a^/c.808C > T		chr17	5253769	5253769	C	T	0,1,1	0,0,0
RABEPK	NM_001174152.1:p.Arg113^a^/c.337C > T	rs199553121	chr9	127975774	127975774	C	T	0,1,1	0,0,0
RGS11	NM_183337.2:p.Arg133^a^/c.397C > T	rs149201684	chr16	324075	324075	G	A	0,1,1	0,0,0
RNF133	NM_139175.1:p.Arg240^a^/c.718C > T	rs141697772	chr7	122338255	122338255	G	A	0,1,1	0,0,0
RNPC3	NM_017619.3:p.Gln185^a^/c.553C > T		chr1	104078061	104078061	C	T	0,1,1	0,0,0
RPGRIP1	NM_020366.3:p.Arg52^a^/c.154C > T	rs192003551	chr14	21762904	21762904	C	T	0,1,1	0,0,0
RPTN	NM_001122965.1:p.Arg771^a^/c.2311C > T	rs192865821	chr1	152127264	152127264	G	A	0,1,1	0,0,0
SCAND1	NM_033630.2:p.Trp43^a^/c.128G > A		chr20	34542376	34542376	C	T	0,1,1	0,0,0
SCD5	NM_024906.2:p.Gln192^a^/c.574C > T		chr4	83582226	83582226	G	A	0,1,1	0,0,0
SCUBE2	NM_001170690.1:p.Cys548^a^/c.1644C > A		chr11	9055237	9055237	G	T	0,1,1	0,0,0
SDSL	NM_138432.2:p.Leu280^a^/c.839 T > G		chr12	113875733	113875733	T	G	0,1,1	0,0,0
SFTPD	NM_003019.4:p.Gln80^a^/c.238C > T	rs79085361	chr10	81702597	81702597	G	A	0,1,1	0,0,0
SLC16A4	NM_004696.2:p.Trp482^a^/c.1446G > A	rs114581294	chr1	110906406	110906406	C	T	0,1,1	0,0,0
SLC22A11	NM_018484.2:p.Arg48^a^/c.142C > T	rs35008345	chr11	64323613	64323613	C	T	0,1,1	0,0,0
SLC22A24	NM_001136506.2:p.Arg347^a^/c.1039C > T	rs374095536	chr11	62863494	62863494	G	A	0,1,1	0,0,0
SLC2A5	NM_003039.2:p.Ser291^a^/c.872C > A		chr1	9099872	9099872	G	T	0,1,1	0,0,0
SLC5A8	NM_145913.3:p.Lys183^a^/c.547A > T		chr12	101587548	101587548	T	A	0,1,1	0,0,0
SLC6A18	NM_182632.2:p.Gln249^a^/c.745C > T	rs200802505	chr5	1239577	1239577	C	T	0,1,1	0,0,0
SPATA24	NM_194296.1:p.Gln185^a^/c.553C > T	rs183526939	chr5	138732554	138732554	G	A	0,1,1	0,0,0
SPERT	NM_152719.2:p.Trp135^a^/c.405G > A		chr13	46287565	46287565	G	A	0,1,1	0,0,0
SSPO	NM_198455.2:p.Gln2048^a^/c.6142C > T	rs200402989	chr7	149491941	149491941	C	T	0,1,1	0,0,0
STMND1	NM_001190766.1:p.Gln60^a^/c.178C > T	rs146229126	chr6	17115289	17115289	C	T	0,1,1	0,0,0
TAS2R19	NM_176888.1:p.Arg39^a^/c.115C > T	rs146593308	chr12	11175056	11175056	G	A	0,1,1	0,0,0
TAS2R20	NM_176889.2:p.Trp35^a^/c.104G > A	rs116400924	chr12	11150371	11150371	C	T	0,1,1	0,0,0
TMEM150B	NM_001085488.2:p.Cys45^a^/c.135C > A		chr19	55831820	55831820	G	T	0,1,1	0,0,0
TMEM70	NM_017866.5:p.Arg80^a^/c.238C > T	rs387907070	chr8	74891018	74891018	C	T	0,1,1	0,0,0
TMX4	NM_021156.2:p.Gln69^a^/c.205C > T	rs373356438	chr20	7990934	7990934	G	A	0,1,1	0,0,0
TOR1AIP1	NM_001267578.1:p.Ser50^a^/c.149C > A		chr1	179851786	179851786	C	A	0,1,1	0,0,0
TREX2	NM_080701.3:p.Arg87^a^/c.259C > T	rs141078733	chrX	152710630	152710630	G	A	0,1,1	0,0,0
TRIOBP	NM_001039141.2:p.Arg1025^a^/c.3073C > T		chr22	38121636	38121636	C	T	0,1,1	0,0,0
TSPAN19	NM_001100917.1:p.Gly19^a^/c.55G > T	rs188656791	chr12	85423670	85423670	C	A	0,1,1	0,0,0
TTC22	NM_017904.3:p.Arg342^a^/c.1024C > T	rs2270002	chr1	55251314	55251314	G	A	0,1,1	0,0,0
TTC25	NM_031421.3:p.Glu602^a^/c.1804G > T	rs375330943	chr17	40117478	40117478	G	T	0,1,1	0,0,0
TTLL3	NM_001025930.3:p.Arg704^a^/c.2110C > T	rs115917139	chr3	9874914	9874914	C	T	0,1,1	0,0,0
UBAP1L	NM_001163692.1:p.Glu34^a^/c.100G > T		chr15	65398454	65398454	C	A	0,1,1	0,0,0
UGT1A7	NM_019077.2:p.Tyr81^a^/c.243C > A	rs149618508	chr2	234590826	234590826	C	A	0,1,1	0,0,0
UGT2A1	NM_001252274.2:p.Tyr192^a^/c.576 T > A	rs111696697	chr4	70512787	70512787	A	T	0,1,1	0,0,0
UMODL1	NM_173568.3:p.Trp1379^a^/c.4136G > A	rs376098587	chr21	43549884	43549884	G	A	0,1,1	0,0,0
UPK3A	NM_006953.3:p.Ser87^a^/c.260C > A	rs138918236	chr22	45683104	45683104	C	A	0,1,1	0,0,0
UTS2B	NM_198152.3:p.Arg111^a^/c.331C > T	rs16866426	chr3	190993044	190993044	G	A	0,1,1	0,0,0
VSIG10L	NM_001163922.1:p.Gly340^a^/c.1018G > T		chr19	51843858	51843858	C	A	0,1,1	0,0,0
VWCE	NM_152718.2:p.Gln866^a^/c.2596C > T	rs61729958	chr11	61026419	61026419	G	A	0,1,1	0,0,0
WDR33	NM_001006622.2:p.Trp265^a^/c.794G > A		chr2	128522234	128522234	C	T	0,1,1	0,0,0
WDR49	NM_178824.3:p.Ser330^a^/c.989C > A		chr3	167250675	167250675	G	T	0,1,1	0,0,0
WRN	NM_000553.4:p.Arg369^a^/c.1105C > T	rs17847577	chr8	30938648	30938648	C	T	0,1,1	0,0,0
ZBED6CL	NM_138434.2:p.Gln29^a^/c.85C > T	rs73474332	chr7	150027578	150027578	C	T	0,1,1	0,0,0
ZIM3	NM_052882.1:p.Lys438^a^/c.1312A > T	rs111350153	chr19	57646393	57646393	T	A	0,1,1	0,0,0
ZNF107	NM_001282359.1:p.Ser181^a^/c.542C > G	rs200723270	chr7	64167017	64167017	C	G	0,1,1	0,0,0
ZNF135	NM_007134.1:p.Glu221^a^/c.661G > T	rs148932599	chr19	58578441	58578441	G	T	0,1,1	0,0,0
ZNF154	NM_001085384.2:p.Arg192^a^/c.574C > T	rs74939505	chr19	58213743	58213743	G	A	0,1,1	0,0,0
ZNF200	NM_003454.3:p.Arg392^a^/c.1174C > T	rs138531369	chr16	3273906	3273906	G	A	0,1,1	0,0,0
ZNF211	NM_001265597.1:p.Tyr602^a^/c.1806 T > A	rs146505315	chr19	58153465	58153465	T	A	0,1,1	0,0,0
ZNF665	NM_024733.3:p.Gln154^a^/c.460C > T	rs74974920	chr19	53669283	53669283	G	A	0,1,1	0,0,0
ZNF718	NM_001289930.1:p.Gln278^a^/c.832C > T	rs116083456	chr4	155530	155530	C	T	0,1,1	0,0,0
ZNF781	NM_152605.3:p.Arg53^a^/c.157C > T	rs140682866	chr19	38160893	38160893	G	A	0,1,1	0,0,0
ZSCAN9	NM_001199479.1:p.Arg193^a^/c.577C > T	rs76542212	chr6	28198122	28198122	C	T	0,1,1	0,0,0
More than one allele in PPROM Cohort									
ABCB5	NM_001163941.1:p.Arg353^a^/c.1057C > T	rs150279505	chr7	20687233	20687233	C	T	0,2,2	0,0,0
ACSM3	NM_005622.3:p.Trp292^a^/c.875G > A	rs52817836	chr16	20792388	20792388	G	A	0,2,2	0,0,0
ALPK1	NM_001102406.1:p.Trp595^a^/c.1785G > A	rs116802171	chr4	113352488	113352488	G	A	0,2,2	0,0,0
AMZ1	NM_001284355.1:p.Arg292^a^/c.874C > T	rs55919423	chr7	2752059	2752059	C	T	0,4,4	0,0,0
AOAH	NM_001177506.1:p.Gln556^a^/c.1666C > T	rs145455591	chr7	36554130	36554130	G	A	0,2,2	0,0,0
C9orf129	NM_001098808.1:p.Gln170^a^/c.508C > T	rs115115786	chr9	96080763	96080763	G	A	0,3,3	0,0,0
CLEC6A	NM_001007033.1:p.Ser200^a^/c.599C > A	rs114953954	chr12	8630029	8630029	C	A	0,2,2	0,0,0
COL6A6	NM_001102608.1:p.Gly1434^a^/c.4300G > T	rs140872639	chr3	130311412	130311412	G	T	0,2,2	0,0,0
COQ6	NM_182480.2:p.Trp14^a^/c.41G > A	rs17094161	chr14	74416836	74416836	G	A	0,6,6	0,0,0
EFCAB5	NM_198529.3:p.Gln952^a^/c.2854C > T	rs73274829	chr17	28405349	28405349	C	T	0,2,2	0,0,0
ERVMER34–1	NM_001242690.1:p.Trp68^a^/c.204G > A	rs61731313	chr4	53611484	53611484	C	T	0,2,2	0,0,0
HSD17B13	NM_178135.4:p.Trp150^a^/c.450G > A	rs61748262	chr4	88238244	88238244	C	T	0,2,2	0,0,0
HTR4	NM_001286410.1:p.Lys389^a^/c.1165A > T	rs58336229	chr5	147861104	147861104	T	A	0,2,2	0,0,0
KCNMB3	NM_014407.3:p.Trp106^a^/c.317G > A	rs145138176	chr3	178962425	178962425	C	T	0,2,2	0,0,0
KRT74	NM_175053.3:p.Gln285^a^/c.853C > T	rs147781415	chr12	52964608	52964608	G	A	0,2,2	0,0,0
MAGEE2	NM_138703.4:p.Glu120^a^/c.358G > T	rs1343879	chrX	75004529	75004529	C	A	1,0,2	0,0,0
MATK	NM_002378.3:p.Arg9^a^/c.25C > T	rs74830030	chr19	3789321	3789321	G	A	0,2,2	0,0,0
METTL7B	NM_152637.2:p.Arg224^a^/c.670C > T	rs115687886	chr12	56077768	56077768	C	T	0,7,7	0,0,0
MROH2B	NM_173489.4:p.Trp191^a^/c.572G > A	rs1023840	chr5	41061715	41061715	C	T	0,3,3	0,0,0
MUC19	NM_173600.2:p.Gln8113^a^/c.24337C > T	rs75211948	chr12	40961512	40961512	C	T	0,3,3	0,0,0
OPRM1	NM_001008503.2:p.Arg401^a^/c.1201C > T	rs34427887	chr6	154567863	154567863	C	T	0,2,2	0,0,0
OR10Z1	NM_001004478.1:p.Tyr153^a^/c.459C > A	rs148998855	chr1	158576687	158576687	C	A	0,3,3	0,0,0
OR6C6	NM_001005493.1:p.Leu200^a^/c.599 T > A	rs76796682	chr12	55688418	55688418	A	T	0,2,2	0,0,0
PHF19	NM_001009936.2:p.Gln180^a^/c.538C > T	rs112858270	chr9	123632050	123632050	G	A	0,3,3	0,0,0
PKD1L2	NM_052892.3:p.Trp1184^a^/c.3551G > A	rs147079883	chr16	81194437	81194437	C	T	1,1,3	0,0,0
PLCXD2	NM_001185106.1:p.Trp292^a^/c.876G > A	rs77085054	chr3	111451475	111451475	G	A	0,2,2	0,0,0
RNF212	NM_001193318.2:p.Gln188^a^/c.562C > T	rs60035268	chr4	1087487	1087487	G	A	0,4,4	0,0,0
SLC10A5	NM_001010893.2:p.Leu201^a^/c.602 T > A	rs112999969	chr8	82606606	82606606	A	T	0,2,2	0,0,0
TAS2R46	NM_176887.2:p.Gln288^a^/c.862C > T	rs150894148	chr12	11214032	11214032	G	A	0,2,2	0,0,0
TCHHL1	NM_001008536.1:p.Gln294^a^/c.880C > T	rs61749316	chr1	152059278	152059278	G	A	0,4,4	0,0,0
ULBP3	NM_024518.1:p.Glu110^a^/c.328G > T	rs34672740	chr6	150387059	150387059	C	A	0,2,2	0,0,0
ZAN	NM_003386.2:p.Trp1883^a^/c.5649G > A	rs2293766	chr7	100371358	100371358	G	A	1,4,6	0,0,0
ZNF486	NM_052852.3:p.Tyr210^a^/c.630C > G	rs184976796	chr19	20308149	20308149	C	G	0,2,2	0,0,0
ZNF594	NM_032530.1:p.Glu684^a^/c.2050G > T	rs114754534	chr17	5085502	5085502	C	A	0,2,2	0,0,0
ZP4	NM_021186.3:p.Arg252^a^/c.754C > T		chr1	238050156	238050156	G	T	0,2,2	0,0,0

WES (50–100 X coverage) data were analyzed as described in (Modi et al., 2017a,b) to extract nonsense variants

All nonsense variants were identified by WES and only selected variants were confirmed by Sanger sequencing

*Chr* chromosome

^a^Allele count: Homozygous, Heterozygous, Total Alleles

A heterozygous nonsense variant (rs5743490) of African ancestry in the Defensin Beta 1 (*DEFB1)* gene, which encodes a small cysteine-rich cationic peptide that damages the cellular membranes of bacteria and some viruses, was found in PPROM cases in our initial WES and targeted genotyping [9], but not in neonates born at term (Tables 1, 2, 3 and 4). No other loss of function variants, including splicing variants and frameshift variants, were identified in *DEFB1* in our WES. DEFB1 is expressed by the fetal membranes [9] (Additional file 2: Figure S1).

**Table 2:** SNPs Evaluated

	rs Number	Nucleotide Change	Protein Sequence Change	Ancestry	MAF
*DEFB1*	rs5743490	G/T	p.Cys37Ter	African	0.0008586
*MBL2*	rs74754826	C/A	p.Glu210Ter	African	0.000594
*METTL7B*	rs115687886	C/T	p.Arg224Ter	African	0.004004
	rs138407179	G/T	p.Gly80Ter	African	7.941e-05
	rs146636131	G/T	p.Arg224Leu	African	0.0005122

*MAF* Minor allele frequency from gnomAD

**Table 3:** Putative Ancestry-Specific Variants Conferring Risk of PPROM

	*DEFB1* rs5743490	*MBL2* rs74754826	*METTL7B* rs115687886
Present in cases only in WES?	Yes^a^	Yes^a^	Yes
Validated by Sanger sequencing?	Yes^a^	Yes^a^	Yes
Rare?	Yes	Yes	Yes
African ancestry?	Yes	Yes	Yes
Under selective pressure?	Yes	Yes	?
Heterozygous impact?	Plausible	Plausible	?
Expressed in fetal membranes?	Yes	Yes^a^	Yes
Plausible pathophysiology?	Yes	Yes	?
Replication?	Yes	Yes	No

^a^Data derived from the present report and Modi et al. [9]

**Table 4:** Allele Counts and Minor Allele Frequencies

rs Number	Term				PPROM				
	MAC/TA	Homo	Het	MAF	MAC/TA	Homo	Het	MAF	*p* Value (nominal)
rs5743490 Combined^a^	1/751	0	1	0.0013	10/694	1	8	0 .0144	(*p* < 0.004)
rs74754826 Combined^a^	1/751	0	1	0.0013	8/694	1	8	0.0115	(*p* < 0.015)
rs115687886 Combined	10(9)/254	1	8(7)	0.035	20(19)/318	0	20(19)	0.060	(*p* > 0.05)

*MAC/TA* Minor allele count/total alleles, *MAF* Minor allele frequency, *Homo* Homozygous, *Het* Heterozygous

(9) = rs115687886 nonsense mutations subtracting out those with an adjacent rs1466636131 minor allele

^a^ Data derived from the present study and Modi et al. [9]

The *DEFB1* rs5743490 SNP has two alternative minor alleles, C/T (African ancestry), which creates a stop codon; and G/A (Latino ancestry), which produces a synonymous amino acid change. We verified by Sanger sequencing that the minor allele of rs5743490 that we detected encoded a stop codon [9]. This *DEFB1* nonsense variant truncates the DEFB1 protein 4 amino acids into the mature peptide amino acid sequence so that no functional DEFB1 would be made [10]. However, the mutant protein, if expressed, could have dominant negative activity by preventing proteolytic processing of the un-mutated pro-peptide encoded by the normal allele. Thus, heterozygous mutations could possibly be functionally significant.

An additional 115 PPROM cases and 191 controls were subsequently genotyped for the *DEFB1* nonsense mutation, yielding more nonsense mutations in PPROM cases, including a neonate with a homozygous *DEFB1* nonsense variant, and only one mutant allele in a term control (Table 4). A statistically significant association of the rs5743490 nonsense mutation and PPROM was present in the combined cohorts (Table 4) (*p* < 0.004 by Fisher’s Exact test, 1-tailed).

We discovered another rare nonsense variant of African ancestry (rs74754826) in the *MBL2* gene, which encodes mannose binding lectin-2, a protein involved in anti-microbial host defense [9]. It was only detected in PPROM cases (WES and initial targeted genotyping), and it met the screening criteria for being a PPROM candidate gene (Tables 1, 2 and 3). One hundred and nineteen PPROM cases and 199 term controls were genotyped in the present study for this nonsense variant, and a statistically significant association of the minor allele with PPROM was found (*P* < 0.015 by Fisher’s Exact test, 1-tailed) (Table 4).

We then applied our WES screening approach to look for other PPROM candidate genes, including genes where the nonsense mutation was of relatively high allele frequency in PPROM cases. We detected 7 heterozygous nonsense variants in the Methyltransferase Like 7B (*METTL7B)* gene in the WES discovery panel in PPROM cases, and none in term controls. This was the largest number of unique “PPROM mutation alleles”. *METTL7B* transcripts were detected in human placenta and amnion by PCR with sequence verification of the amplicon (Additional file 2: Figure S1).

A *METTL7B* SNP (rs146636131) adjacent to rs115687886 that is in phase modifies the codon to create a benign missense variant (p.Arg224Leu). Subjects with both rs115687886 and rs146636131 minor alleles were considered to have the missense variant rather than the nonsense mutation (Tables 1, 2, 3 and 4). Another rarer nonsense variant (rs138407179) was also detected. Both *METTL7B* nonsense variants are identified as causing loss of function with “high confidence” in the gnomAD database. rs138407179 has two alternate minor alleles, one encoding the stop codon of African ancestry (G/T) and another (G/A), which encodes a predicted damaging variant of South Asian ancestry.

Follow-up targeted genotyping of the *METTL7B* SNPs of interest on 94 PPROM cases and 94 term controls detected the nonsense variant in term controls, including a homozygote mutant. Statistical analysis of the combined analysis WES data and follow-up genotyping revealed no statistically significant association of the *METTL7B* rs115687886 nonsense mutation with PPROM (Tables 3 and 4), a finding that was not unexpected based on the fact that the minor allele of rs115687886 did not robustly meet all screening criteria as noted above.

## Discussion

The simple screening approach outlined above may be useful to others seeking rare variants with moderate to high effect size associated with preterm birth in specific populations. The approach can also be used to identify rare mutations that are protective for PPROM by starting the screening with selection of variants found only in term controls, not in PPROM, and applying the subsequent filters. The fact that the majority of WES nonsense variants were detected in a single PPROM case, but each individual case harbored multiple nonsense variants allows for a test of genetic burden to be conducted as we have done in our previous studies [8, 9] in addition to the more focused examination of the contributions of individual variants. Importantly, the patterns of nonsense mutations among PPROM cases and term controls could also point to pathways and gene networks that when disrupted promote PPROM or protect against it.

Our findings suggest that a rare damaging *DEFB1* variant of African ancestry may have a role in the pathophysiology of PPROM, presumably because it facilitates a dysbiotic reproductive tract flora that invades and or inflames the fetal membranes leading to premature rupture. The *DEFB1* gene has been under selective pressure [11, 12], and has rare loss of function variants with four different ancestries (African, Latino, East Asian, European) reported in the genomAD database. It will be of interest to determine in the future if the other *DEFB1* loss of function variants play a role in preterm birth after PPROM in the respective populations.

The discovery that the *DEFB1* nonsense variant is associated PPROM prompted us to examine damaging variants in other beta defensin genes in our WES study. A stop-loss variant of African ancestry was identified in *DEFB119* (rs12329612) in 19 PPROM cases, including 1 homozygote (20 alleles/152 total alleles), and 5 term controls, including 1 homozygote. A start-loss variant detected in *DEFB128* (rs145944118) was found in one PPROM case, and another start-loss variant (rs18818350) was detected in *DEFB132* in one term control. The functional significance of these variants and their relationship to preterm birth are currently unknown.

We discovered a significant association between a nonsense variant in an anti-microbial gene, *MBL2*, and PPROM. This association is consistent with the work of others who examined common *MBL2* polymorphisms in fetal DNA from European populations and found increased risk of preterm birth [13, 14]. MBL-2 presumably functions as part of the host defense system, including DEFB1, which prevents or limits infections that cause chorioamnionitis and PPROM.

In contrast to our findings with the *DEFB1* and *MBL2* nonsense variants, the nonsense variant (rs115687886) in the *METTL7B* gene did not stand up to further scrutiny as a PPROM candidate. *METTL7B* encodes a putative methyltransferase whose transcript is elevated in blood leukocytes in the context of infection in pregnancy, providing a potential link to PPROM mechanisms [15]. However, the METTL7B was initially reported to be a lipid droplet-associated protein whose function with respect to lipid metabolism remains obscure [16].

The *METTL7B* rs115687886 minor allele (in the absence of the adjacent in phase SNP) truncates the protein at amino acid position 224 of the 244-amino acid protein. This truncation is outside of the methyltransferase domain (amino acids 75–172). The functional significance of this protein truncation has not been established to the best of our knowledge, which could make the mutation “ineligible” in our screening criteria. Moreover, the rs115687886 minor allele frequencies in our African-American term controls and PPROM cases are relatively high (Table 4) and outside of our definition of “rare” (Allele frequency < 0.01). No splicing or frameshift variants predicted to disrupt the protein coding sequence were detected in *METTL7B* the WES sample.

The other nonsense minor allele we examined (rs138407179), found in both a PPROM case and a term control, truncates the protein at amino acid residue 80, which likely damages the protein. Genotyping of additional PPROM cases and controls is required to determine if this nonsense variant is associated with PPROM. We could find no information in the literature regarding selective pressures impinging on the *METTL7B* gene.

Although our proposed strategy is consistent with guidelines for investigating causality of sequence variants in human disease, our approach has limitations including the current cost of WES and the use of modest sample sizes which may not have the power to detect pathophysiologic important rare variants/mutations [17]. The focus on nonsense variants found only in cases might exclude important PPROM-associated mutations from consideration if there was by chance a nonsense variant in the control group but not the cases. Importantly, the analysis strategy used in this report did not encompass other potentially damaging variants including frameshift, splicing and damaging missense variants, including variants that cause gain of function. These variants/mutations could, of course, be incorporated into the screening algorithm. In addition, WES would not identify intragenic regulatory elements that have an impact on gene expression levels. Another limitation of our study is the absence of direct evidence for disrupted function of the DEFB1 and MBL-2 proteins derived from the respective mutant transcripts. That said, the *DEFB1* nonsense mutation would not lead to production of a mature peptide, so it is most certainly damaging. However, its potential to be a dominant negative inhibiting processing of full length DEFB1 pro-peptide from the major allele in heterozygous mutants remains to be explored. Likewise, the impact of the *MBL2* nonsense mutation on protein function is only predicted, and studies need to be conducted with recombinant proteins to prove loss of function.

Our findings on the *DEFB1* and *MBL2* nonsense mutations are consistent with the notion that rare fetal mutations contribute to the disparities in preterm birth among African-Americans, and support the mining of rare mutations identified in WES as a portal to discovery of genes playing a role in preterm birth. A similar approach could be applied to other populations focusing on ancestry-enriched damaging variants. For example, there are rare damaging Latino (rs759177517; p. Tyr5Ter) and East Asian (rs140403947, p. Tyr60Ter) nonsense variants in the *DEFB1* gene that could be evaluated for association with PPROM in the respective populations.

## Conclusions

Our findings based on a simple and cost-effective data analysis strategy support the notion that multiple rare population-specific variants in the fetal genome contribute to preterm birth associated with PPROM.

## Additional files

Additional file 1: **Table S1.** Primers used for *METTL7B* SNP genotyping. Primers used for genotyping rs115687886, rs138407179, rs146636131. (DOCX 11 kb)

Additional file 2: **Figure S1.** RT-PCR analysis for expression of *DEFB1* and *METTL7B*. A. The first well shows the expression of *DEFB1* in a sample from amnion and the second well a sample from placental tissue. A negative control was loaded in the third well. The primers used for the transcript amplification were forward 5’-CTGAAATCCTGGGTGTTGCC-3′ and reverse 5′- CTTCTGGTCACTCCCAGCTC- 3′. Amplified bands were sequence verified. B. The first well shows *MBL2* expression in amnion and the second well a sample from placental tissue. A negative control was loaded in the third well. The primers used for the transcript amplification were forward 5’-ACCTGCCTAGACCCAAATCC-3′ and reverse 5′- TTATTTGACAGCCTTTCCCATGA-3′. Amplified bands were sequence verified. (TIF 789 kb)

## Abbreviations

DEFB1: Defensin beta 1
MBL2: Mannose-binding lectin-2
METTL7B: Methyltransferase like 7B
PPROM: Preterm premature rupture of membranes
WES: Whole exome sequencing
