# Supplementary material for: Discovery of rare ancestry-specific variants in the fetal genome that confer risk of preterm premature rupture of membranes (PPROM) and preterm birth
Source: BMC Med Genet. 2018 Oct 5;19:181. doi: 10.1186/s12881-018-0696-4 (PMC6173941; doi:10.1186/s12881-018-0696-4)
Supplement: Supplementary file 1 — Table S1. Primers used for METTL7B SNP genotyping. Primers used for genotyping rs115687886, rs138407179, rs146636131. (DOCX 11 kb) [file 12881_2018_696_MOESM1_ESM.docx]

**Table S1 Primers used for *METTL7B* SNP genotyping**

**rs115687886:** AACACATTGGGGATGGCTGCTGCCTCACCAGAGAGACCTGGAAGG**[**A/G/T**]**TCTTGAGAACGCCCAGTTCTCCGAAATCCAAATGGAA**[**C/T**]**G

**rs138407179:**

CTTACAGGAGCCTCCGGGAAAGTGGCCCTACTGGAGCTGGGCTGC**[**A/G/T**]**GAACCGGAGCCAACTTTCAGTTCTACCCACCGGGCTGCAGGGT

**rs146636131:**

GGAAGGATCTTGAGAACGCCCAGTTCTCCGAAATCCAAATGGAAC**[**A/G/T**]**ACAGCCCCCTCCCTTGAAGTGGCTACCTGTTGGGCCCCACATC
