# Supplementary figures and images for: Discovery of rare ancestry-specific variants in the fetal genome that confer risk of preterm premature rupture of membranes (PPROM) and preterm birth
Source: BMC Med Genet. 2018 Oct 5;19:181. doi: 10.1186/s12881-018-0696-4 (PMC6173941; doi:10.1186/s12881-018-0696-4)

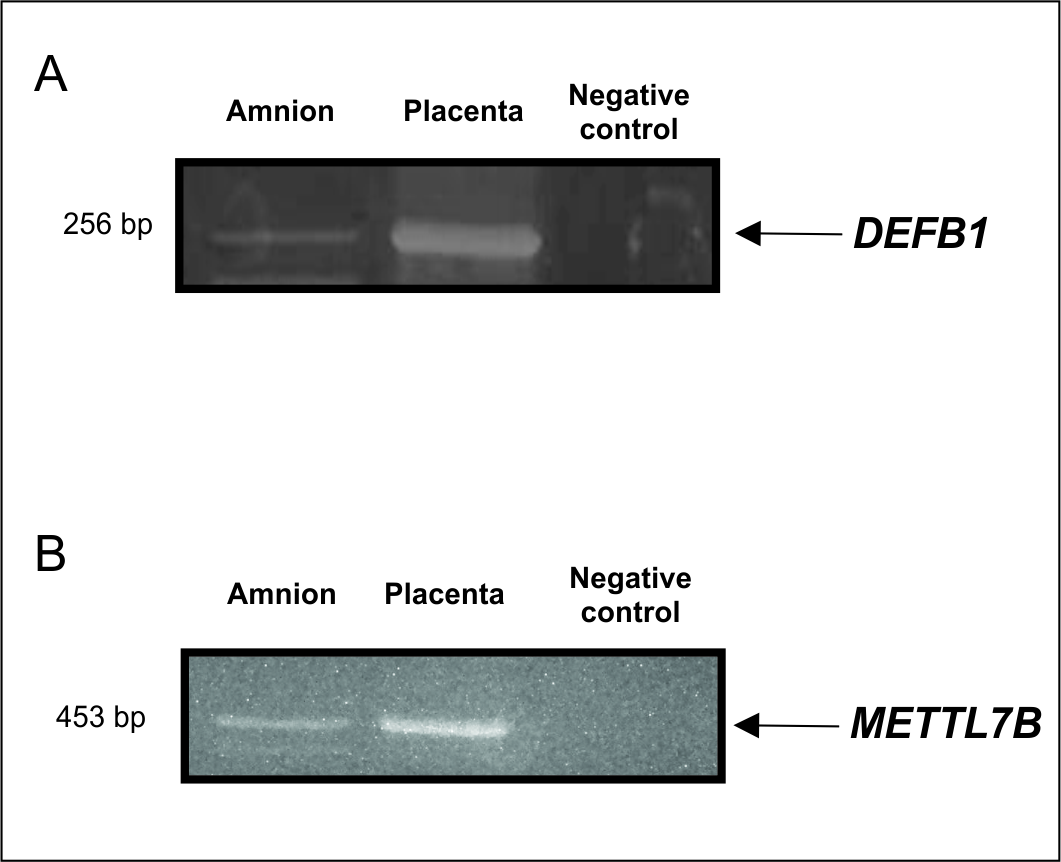

Supplement: Supplementary file 2 — Figure S1. RT-PCR analysis for expression of DEFB1 and METTL7B. A. The first well shows the expression of DEFB1 in a sample from amnion and the second well a sample from placental tissue. A negative control was loaded in the third well. The primers used for the transcript amplification were forward 5’-CTGAAATCCTGGGTGTTGCC-3′ and reverse 5′- CTTCTGGTCACTCCCAGCTC- 3′. Amplified bands were sequence verified. B. The first well shows MBL2 expression in amnion and the second well a sample from placental tissue. A negative control was loaded in the third well. The primers used for the transcript amplification were forward 5’-ACCTGCCTAGACCCAAATCC-3′ and reverse 5′- TTATTTGACAGCCTTTCCCATGA-3′. Amplified bands were sequence verified. (TIF 789 kb) [file 12881_2018_696_MOESM2_ESM.tif]
